# Herb-Induced Liver Injury (HILI) Associated With Asowosi (Momordica charantia): A Case Report

**DOI:** 10.7759/cureus.101682

**Published:** 2026-01-16

**Authors:** Daniela Guerra, Elham Shams, Pedro Igorra, Sarah Nickle, Muhammad Aziz

**Affiliations:** 1 Medicine, Herbert Wertheim College of Medicine, Florida International University, Miami, USA; 2 Internal Medicine, Jackson Memorial Hospital, Miami, USA; 3 Translational Medicine, Herbert Wertheim College of Medicine, Florida International University, Miami, USA; 4 Internal Medicine, Herbert Wertheim College of Medicine, Florida International University, Miami, USA

**Keywords:** asowosi, bitter melon, dietary supplements, herbal hepatotoxicity, naturopathy, transaminitis

## Abstract

Herbal remedies are widely used for various health purposes, yet their potential to cause adverse effects is often overlooked. Asowosi, also known as bitter melon, is one such plant incorporated into traditional medicine practices for blood sugar management.

This case study presents the comprehensive findings regarding a 38-year-old male who initially presented with transaminitis and weakness. The cause of the patient’s elevated liver enzymes was unclear, as he reported occasional alcohol consumption. However, upon further questioning, he disclosed two to three weeks of herbal remedy use, including Asowosi. The supplement had been selected for its accessibility and familiarity. While the exact mechanism of injury in this case remains unclear, herbal products can have physiologic effects that warrant consideration during diagnostic evaluation.

Determining the etiology of this patient’s acute liver injury was challenging due to the limited clinical information available on Asowosi. After supportive care and discontinuation of the herbal remedies, the patient’s condition improved, and liver histology supported a diagnosis of herb-induced liver injury (HILI). This case underscores the importance of including herbal and nonprescription products in the differential diagnosis of unexplained liver injury and encourages prompt recognition and reporting of similar cases.

## Introduction

Drug-induced liver injury (DILI), more specifically herb-induced liver injury (HILI), is a rare and potentially serious adverse reaction that can range clinically from asymptomatic transaminitis to acute liver failure. The use of herbal and dietary supplements (HDS) has been increasing both in the United States and worldwide, and HDS-induced liver injury has thus increased proportionally [1]. Traditional medicine is widely used in developing countries due to its accessibility, affordability, and alignment with cultural practices, often serving as an alternative to Western medicine [2]. The most common indications for HDS use include obesity/weight loss, bodybuilding, menopausal symptoms, gastrointestinal disorders, such as indigestion or constipation, liver disease, and migraines [3].

The mechanism of hepatotoxicity from these herbs and supplements is poorly understood [2,3]. Around 70% of patients who sustain liver injury attributable to HDS manifest a hepatocellular pattern of injury. Some herbs and supplements have been documented to cause direct toxic effects, while others have been shown to cause immune-mediated damage within hepatocytes. Known compounds, such as pyrrolizidine alkaloids found in herbs like comfrey, can cause direct hepatocyte damage, leading to necrosis or apoptosis [3]. Additionally, immune-mediated injury may arise when compounds, such as haptens, provoke an inflammatory response, resulting in hepatocyte damage. Most cases of HILI are idiosyncratic, meaning they occur unpredictably, independent of dose, and are influenced by individual factors, such as age, gender, metabolism, immune responses, coexisting diseases, inflammation, co-exposures, and nutritional status [2]. This idiosyncratic mechanism was evident in our patient, who developed acute hepatocellular injury despite no prior liver disease, no clear dose-response relationship, and a temporal association with herbal supplement exposure. This unpredictability further adds to the difficulty in both the diagnosis and management of HILI.

While many herbs and supplements are well-documented hepatotoxins, numerous others with potential side effects remain insufficiently studied. The complexity of herbal remedies, which often contain a combination of active compounds, additives, and contaminants, complicates the identification of specific agents responsible for liver damage. These factors, combined with physician unawareness and patients’ reluctance to disclose their use of herbal supplements due to fear of judgment or the belief that these products are harmless, contribute to the widespread underreporting and underdiagnosis of HILI [4]. As a result, consumers are placed at risk, often unaware of the potential dangers posed by these seemingly benign remedies.

In this report, we present a case of a 38-year-old male who developed hepatotoxicity in the setting of alcohol misuse and the concurrent use of multiple “natural remedies,” including Asowosi. Asowosi is derived from bitter melon, a plant known for its bioactive compounds, such as peptides and glycosides, which are believed to enhance insulin sensitivity and lower blood sugar levels. However, while these compounds offer therapeutic benefits, they may also pose a risk to liver health, potentially exerting toxic effects.

## Case presentation

Initial presentation

A 38-year-old male with a history of hypertension and hyperglycemia, without a controlled regimen aside from herbal supplement use, presented to the Emergency Department with a one-week history of epigastric abdominal discomfort worsened with eating, mild odynophagia, generalized weakness, and poor appetite. At the time of presentation, he denied nausea, vomiting, or diarrhea, but noted intermittent fever and "cold-like" symptoms. The patient reported using a blend of supplements containing Asowosi, cactus, and aloe vera, which he had obtained from Haiti and consumed twice daily for two to three weeks before admission, for glycemic control and symptom management. He also admitted to taking amoxicillin and nimesulide for a recent “chest infection.” He recalled a prior episode of abnormal liver function tests in Haiti several years ago, which was self-limited and treated symptomatically. He reported occasional alcohol use, consuming two to three drinks per week, and a family history of a sister who died from liver-related complications.

Clinical course and diagnostic workup

The patient complained of generalized weakness, abdominal pain, and loss of appetite, without nausea, vomiting, or diarrhea. On physical examination, he had a distended abdomen, left lower-quadrant tenderness, and a clinically palpable spleen, with normoactive bowel sounds. However, subsequent abdominal ultrasound did not confirm splenomegaly, and there was no sonographic evidence of portal hypertension, including the absence of ascites, normal portal vein caliber, and preserved hepatopetal flow. Upon admission, the patient was mildly hypertensive, and laboratory studies demonstrated transaminitis, with aspartate aminotransferase (AST) 176 U/L, alanine aminotransferase (ALT) 163 U/L, alkaline phosphatase (ALP) 600 U/L (mixed pattern), and a total bilirubin of 2.8 mg/dL. In addition, the patient had hypoalbuminemia (2.8 g/dL) and mild anemia (Hb 11.8 g/dL). His glucose was elevated at 197 mg/dL, with an HbA1c of 8.9%, confirming diabetes mellitus. An abdominal ultrasound revealed hepatic steatosis without other significant abnormalities. Ultrasound elastography performed early in the hospital course demonstrated moderate to severe fibrosis (METAVIR F>2).

Clinical workup included considering possible causes such as viral etiologies, autoimmune conditions, genetic conditions, drug toxicity, metabolic conditions, and neoplastic conditions. Viral studies, including hepatitis panels, influenza, RSV, and COVID-19, were negative. Additional labs, including autoimmune panels, were also negative. Iron studies were obtained to investigate hemochromatosis due to the patient's family history of liver disease. The patient's ferritin was elevated at >4000 ng/mL; however, this value is not consistent with hemochromatosis, but likely an acute phase reactant. Additionally, transferrin saturation (>50%) suggested chronic disease. The full set of laboratory results, including biochemical markers, autoantibodies, infectious disease diagnostics, and toxicological parameters, is summarized in Table 1.

**Table 1:** Laboratory results Measured values and reference ranges for biochemical markers, autoantibodies, infectious-disease diagnostics, and therapeutic/toxicological parameters are included.

General Chesmitry Day 1	Report	Ref. Range
Ferritin	4,540.00 ng/mL	17.90 - 464.00
Alpha 1 Anti-trypsin Quant	133 mg/dL	88 - 183
Iron	160 mcg/dL	49 - 181
Immunology (Day 1&2)	Report	Ref. Range
ANA Speckled	Fine Granular	-
ANA Speck Titer	0.097	-
Anti-mitochondrial Ab	Negative	Negative
Anti-SSA	Negative	Negative
Anti-SSB	Negative	Negative
Anti-Smith	Negative	Negative
Anti-RNP	Negative	Negative
Anti-SMRNP Antibody	Negative	Negative
Anti-ribosomal Antibody	Negative	Negative
Anti-scleroderma 70	Negative	Negative
Anti-JO1 Ab	Negative	Negative
Anti-chromatin Ab	Negative	Negative
Anti-centromere Ab	Negative	Negative
Anti-Ds DNA	<1 Int Unit/mL	0-4
Anti-neutrophil cytoplasmic Ab	Negative	Negative
Infectious Disease (Day 2)	Report	Ref. Range
CMV DNA Qnt PCR IU/mL	Not Detected	-
EBV Ab (IgG) to Early Ag	<0.2	Antibody Index 0.0 - 0.8
EBV Ab (IgG) to Nuclear Ag	>8.0	Antibody Index 0.0 - 0.8
EBV Ab (IgG) to Viral Capsid Ag	6.9	Antibody Index 0.0 - 0.8
EBV Ab (IgM) to Viral Capsid Ag	<10.00 IU/mL	0.00 - 35.99
HSV 1 PCR	Not Detected	-
HSV 2 PCR	Not Detected	-
Flu A	Not Detected	-
Flu B	Not Detected	-
RSV	Not Detected	-
Strep A by PCR	Not Detected	-
Hepatitis B Virus DNA Qnt PCR	Not Detected	-
HIV AG/AB	Negative	Negative
SARS-CoV2 RNA, RT PCR	Negative	Negative
Hepatitis B Surface Ag	0.11	Negative
Hepatitis C Ab	0.01	Negative
Therapeutic Drug/Tox Screen	Report	Ref. Range
Acetaminophen Level	<10.0	10.0 - 30.0
PEth 16:0/18:1 (POPEth)	>400	<20

The patient’s liver chemistries initially showed improvement, but subsequently worsened, prompting the initiation of IV N-acetylcysteine (NAC) on hospital day 3. Although NAC is well established as the antidote for acetaminophen toxicity, it is also used in non‑acetaminophen‑associated acute liver injury for its antioxidant and cytoprotective effects, and evidence suggests that intravenous NAC may improve transplant‑free survival and hepatic outcomes in the early stages of non‑acetaminophen acute liver failure by enhancing glutathione replenishment and reducing oxidative stress [5]. This intervention coincided with the stabilization of liver enzymes. He experienced a decline in hemoglobin to 7.6 by hospital day 4, prompting the transfusion of one unit of packed red blood cells (PRBCs).

**Figure 1:**
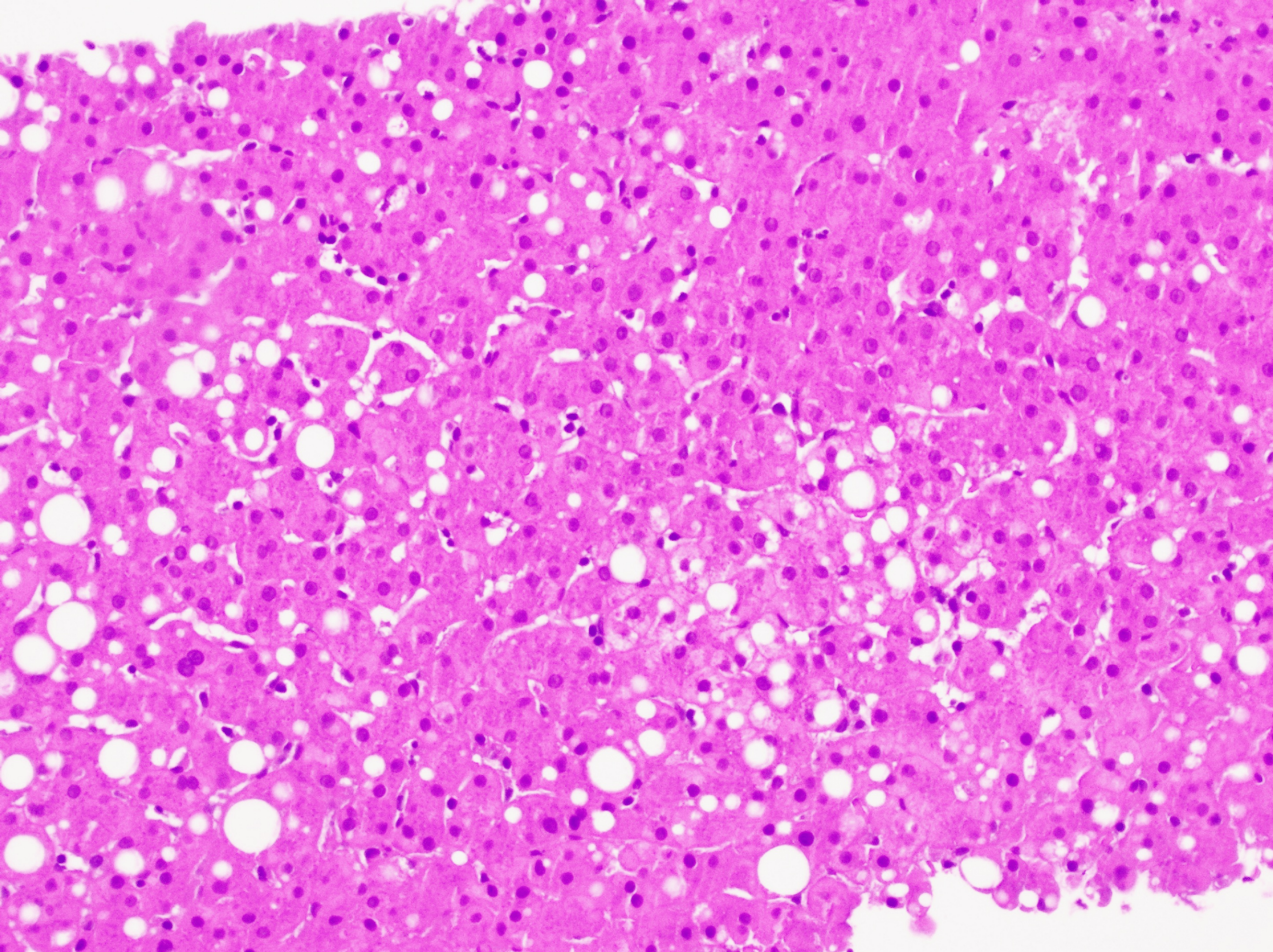
Steatosis on H&E stain

**Figure 2:**
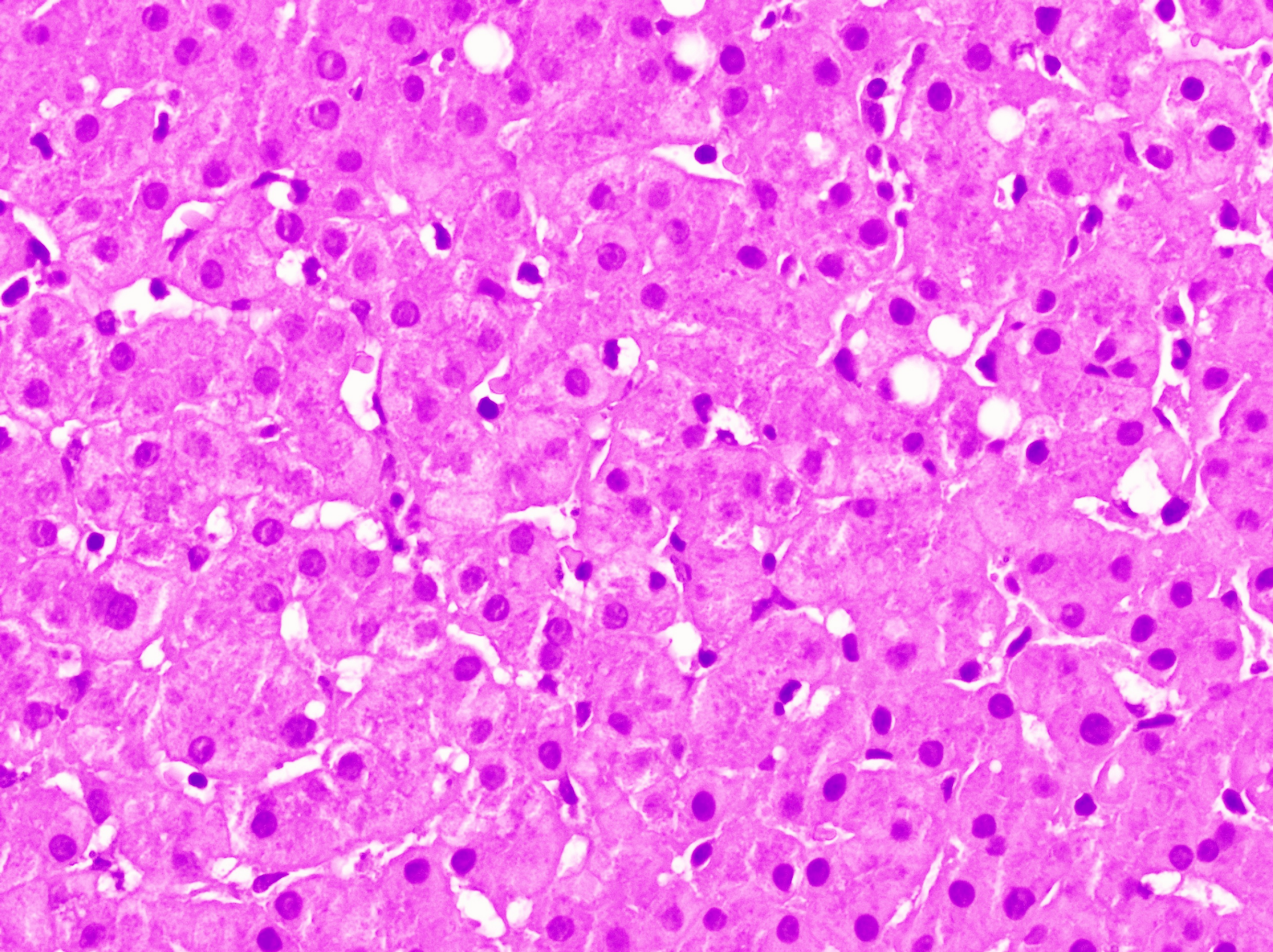
Intracanalicular bile cholestasis on H&E stain

**Figure 3:**
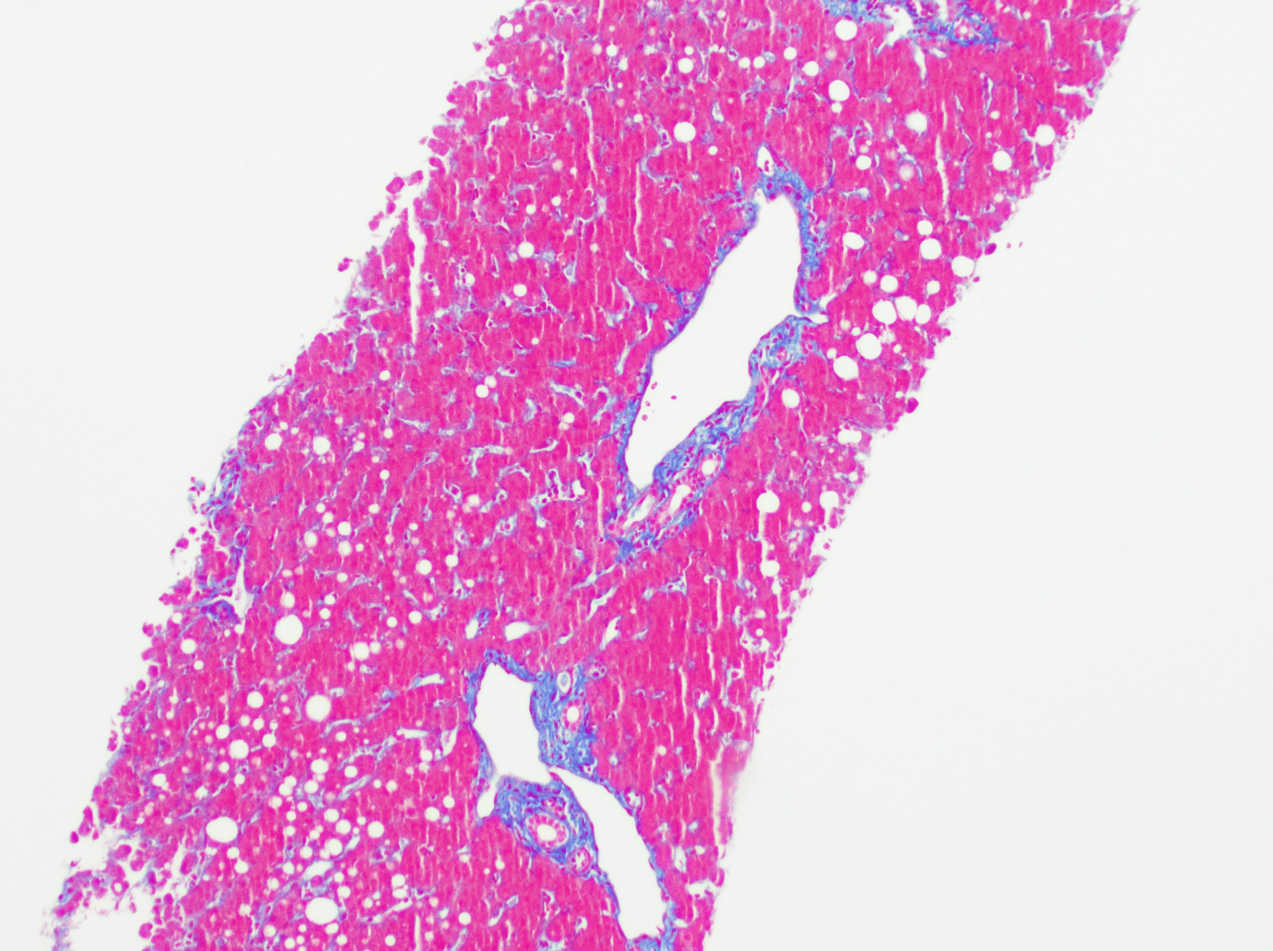
Trichrome showing minimal fibrosis

**Figure 4:**
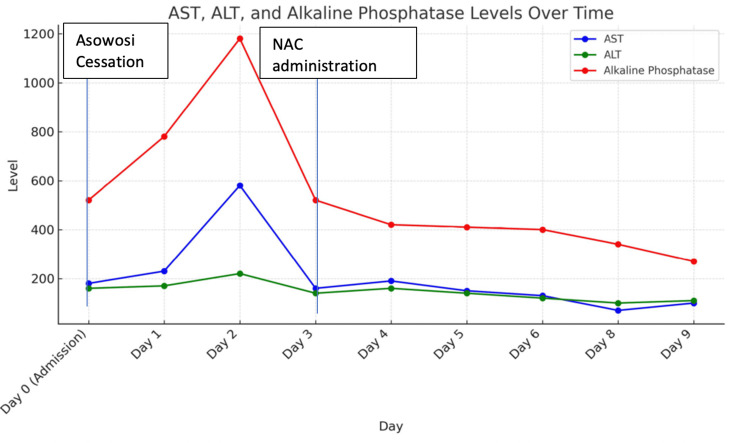
An illustration of the trends in transaminase and ALP levels during the patient's hospital admission AST, Aspartate Aminotransferase; ALT, Alanine Aminotransferase; ALP, Alkaline Phosphatase; NAC, N-acetylcysteine

On hospital day 7, a liver biopsy was performed, and findings revealed mild macrovesicular steatosis (20%) (Figure 1) without ballooned hepatocytes, Mallory-Denk bodies, or mild pericentral cholestasis (Figure 2), and mild iron deposition in Kupffer cells and hepatocytes. There was minimal fibrosis (Figure 3), no significant duct abnormality, or features of alpha-1 antitrypsin deficiency. The histologic findings were consistent with bland cholestasis, suggesting DILI potentially related to herbal supplements, transient choledocholithiasis, or sepsis. However, biliary obstruction was effectively ruled out by a negative right upper-quadrant ultrasound, which showed no gallstones, bile duct dilation, or other structural abnormalities, supporting a hepatocellular or cholestatic injury of non-obstructive origin. Liver enzyme and alkaline phosphatase levels throughout the admission demonstrated an initial worsening, followed by stabilization after NAC initiation (Figure 4).

Management

The patient received IV NAC, which stabilized his liver enzymes. His newly diagnosed diabetes mellitus was managed with insulin therapy (Lantus and sliding scale), with metformin planned for outpatient treatment. Mild electrolyte abnormalities were corrected with IV and oral supplements. Anemia, likely secondary to chronic disease, was managed with a PRBC transfusion. Constipation and abdominal discomfort were addressed with supportive medications, including pantoprazole, laxatives, and anti-nausea agents.

Outcome

By the time of discharge, the patient showed clinical and laboratory improvement, including stabilization of liver enzymes and hemoglobin levels. He was referred for outpatient hepatology and endocrinology for management of chronic liver disease and diabetes mellitus, respectively.

## Discussion

HILI is an increasingly recognized clinical concern, particularly with the growing global use of herbal remedies and dietary supplements. Cases of HDS‑induced liver injury have risen over time, and products of this class now account for approximately 20% of all DILI cases reported in the United States, up from about 7% in the mid‑2000s, and have been implicated in a substantial proportion of acute liver failure cases linked to supplements [6]. These products, though often considered safe and natural, can be associated with significant liver toxicity. Asowosi, derived from the bitter melon plant (*Momordica charantia*), is one such herbal remedy that has been used for various therapeutic purposes, including the management of diabetes in Haiti and other regions. However, data regarding its potential hepatotoxic effects remain sparse [7].

In this case, the patient presented with generalized weakness, epigastric pain, fever, and elevated liver chemistries, which ultimately led to a diagnosis of HILI. A detailed history revealed the use of multiple herbal supplements obtained from Haiti, including Asowosi, cactus, and aloe vera. The combination of these remedies, alongside the patient's history of poorly controlled diabetes and occasional alcohol consumption, was likely a contributing factor to the development of hepatotoxicity. Laboratory evaluation revealed markedly elevated ferritin at >4000 ng/mL, reflecting severe inflammation and hepatocellular injury. Such extreme hyperferritinemia raises the possibility of substantial hepatocyte necrosis, consistent with acute severe HILI. Liver biopsy findings were consistent with DILI, further supporting the diagnosis.

Prompt recognition of the association between Asowosi and liver injury, along with the decision to stop the herbal mixture, resulted in a favorable outcome. However, this case highlights a lack of a well-established evidence base regarding the safety of many herbal remedies. In addition, it is important to note that although nimesulide is a well-recognized cause of DILI, it was considered less likely in this case given the longer duration and consistent twice-daily exposure to Asowosi-containing herbal supplements preceding symptom onset, the temporal relationship between supplement use and liver enzyme elevation, and the absence of recurrent exposure to nimesulide, making the herbal preparation the more probable trigger of hepatotoxicity.

Although data on Asowosi and its association with liver failure are limited, a comparable case was found by Kwentoh et al. (2023) involving a patient who developed jaundice and elevated liver enzymes following Asowosi use in New York City [8]. Similar to our patient, the remedy was perceived as harmless due to its natural origins, but the patient eventually developed severe liver damage, requiring hospitalization [4]. This case further underscores the unpredictable and often idiosyncratic nature of HILI, where the mechanism of toxicity is not well understood and can vary significantly between individuals.

Ultimately, further studies are needed to better understand the liver toxicity of *M. charantia* and similar herbal remedies, ensuring that safety profiles are established to protect consumers from potential harm. Clinicians should routinely ask patients about herbal supplement use using open-ended questions, and patients should be educated that “natural” does not equate to “safe.” Thoroughly conducting patient histories, careful diagnostic evaluations, and heightened awareness of the risks associated with natural remedies are critical for early diagnosis and effective management. Improved regulation and quality control of herbal products, combined with patient education, are necessary steps to mitigate the risks of DILI and ensure safer use of supplements [6].

## Conclusions

HDS-DILI remains an underrecognized but clinically significant cause of hepatotoxicity and can closely mimic other etiologies of liver dysfunction. This case highlights Asowosi (*M. charantia*) as a potential and underreported hepatotoxin, emphasizing that commonly used “natural” remedies, particularly those obtained outside a regulated market, may be associated with clinically meaningful liver injury. It underscores the importance of routinely eliciting a detailed history of herbal and nonprescription supplement use, including internationally sourced products, when evaluating unexplained elevations in liver chemistries.

The diagnostic complexity in this case was further compounded by the presence of multiple potential hepatotoxic exposures, including Asowosi, nimesulide, and alcohol, necessitating a systematic and comprehensive approach to differential diagnosis. Early recognition of HDS-DILI and prompt discontinuation of suspected offending agents were critical in preventing progression to advanced liver injury, reinforcing the need for heightened clinician awareness, patient education regarding supplement-related risks, and improved regulatory oversight to mitigate preventable cases of HDS-associated hepatotoxicity.
